# Efficacy and Safety of Cataract Extraction with Negative Power Intraocular Lens Implantation^§^

**DOI:** 10.2174/1874364100802010015

**Published:** 2008-02-15

**Authors:** Michael A Kapamajian, Kevin M Miller

**Affiliations:** 1 From the Department of Ophthalmology and Visual Sciences, University of Illinois at Chicago Eye and Ear Infirmary, USA; 2 From the Department of Ophthalmology, David Geffen School of Medicine at UCLA and the Jules Stein Eye Institute, Los Angeles, California, USA

## Abstract

**Purpose:**

To evaluate the visual and refractive outcomes, lens power calculation accuracy, and safety of negative power intraocular lenses (IOLs) implanted in highly myopic eyes at the time of cataract surgery.

**Design:**

Interventional case series.

**Methods:**

Sixteen consecutive highly myopic eyes implanted with IOLs from –1 D to –6 D were identified. IOL power; preoperative and postoperative best-corrected visual acuity (BCVA); postoperative uncorrected visual acuity (UCVA); preoperative, intended, and achieved spherical equivalent (SE) refractive errors; and operative complications were recorded.

**Results:**

Median UCVA improved from finger counting to 20/50-2. Median BCVA improved from 20/125-1 to 20/30+1. Mean axial length was 32.65 mm. The mean SE refractive error was –22.19 ± 5.4 D before surgery and -0.28 ± 1.4 D after surgery. The difference between the mean intended and mean achieved SE refractive errors was +1.16 D for the SRK/T, +1.2 D for the Holladay 1, and +1.60 D for the Hoffer Q formulas. Only 5 (33.3%) of 15 eyes in which postoperative measurements were possible were within 1 D of the intended SE postoperative refraction. Postoperative complications included a mildly hyperopic postoperative refractive error (+1.75 D) in one eye necessitating an IOL exchange and posterior capsule opacification in most eyes. There were no retinal detachments.

**Conclusions:**

The SRK/T formula had the greatest accuracy and predictability when immersion A-scan ultrasonography was used to measure axial length. The mean achieved postoperative refractive error was +1.16 D more hyperopic than predicted by this formula. We recommend targeting highly myopic eyes for –1.5 D using the SRK/T formula if a negative power IOL is calculated and emmetropia or mild residual myopia is the desired postoperative result.

## INTRODUCTION

Comorbidities associated with extreme myopia include high astigmatism, posterior staphylomata, myopic retinal degeneration, peripheral retinal thinning, and retinal detachment; and they may reduce the visual benefit of cataract surgery and increase the likelihood of operative complications [1]. The difficulty of measuring the distance from the corneal apex to the functional fovea in the presence of posterior segment staphylomata makes intraocular lens (IOL) power calculation less accurate in extremely myopic eyes [2,3]. Fortunately, the final rest position of an IOL inside an extremely myopic eye is not so critical because minor variations in vertex distance produce relatively insignificant changes in effective IOL power when the actual IOL power is low.

Negative power IOLs have been available for many years through several different manufacturers; however, relatively few have been implanted because of the low prevalence of eyes myopic enough to require one. This retrospective case series evaluates the visual and refractive outcomes of negative power IOL implantation in this patient group, the accuracy and predictability of current IOL power calculation formulas in predicting spherical equivalent (SE) postoperative refractive errors, and the safety of cataract surgery in these eyes.

## PATIENTS AND METHODS

Permission to perform this retrospective study was obtained from the Human Subjects Protection Committee of the UCLA School of Medicine. We identified 16 consecutive eyes from 12 patients using operating room IOL inventory records. All patients underwent cataract extraction with negative power posterior chamber IOL implantation at the Jules Stein Eye Institute between December 1996 and April 2002. All operations were performed by one of the authors (KMM) using the Kelman phacoemulsification technique. Data recorded from each medical record included demographic information; IOL power; preoperative and postoperative best-corrected visual acuity (BCVA); postoperative uncorrected visual acuity (UCVA); preoperative, intended, and achieved spherical equivalent (SE) refractive errors; and operative complications.

Axial lengths were determined by immersion A-scan ultrasonography in all but two eyes. In one of these eyes a B-scan ultrasound technique was used and in the other eye the Humphrey Zeiss IOLMaster (Carl Zeiss Meditec Inc., Jena, Germany) was used. The IOLMaster was not commercially available early in the study when most of the eyes had surgery. IOL powers and predicted postoperative SE refractive errors were calculated using the theoretical Sanders-Retzlaff-Kraff (SRK/T) formula in all cases. This is the default formula for eyes with high axial lengths using the Hoffer power calculator, which we use at the Jules Stein Eye Institute.

The power calculation data sheets in our charts lacked the postoperative SE refractive targets for the Hoffer Q and Holladay 1 equations, which we needed to compare the accuracy and predictability of the three equations. We determined these values by calculating them directly from the respective equations using the measured axial lengths, keratometry values, and published A constants.

Of note, patient 7 required an IOL exchange because of an IOL power calculation error. The patient had a documented staphyloma before surgery. The SRK/T formula was used to calculate IOL power. Her postoperative refraction was +1.75 sphere after cataract surgery and before IOL exchange; this refractive result was used in our analysis of IOL power calculation accuracy. After the implanted –1 D Staar AQ5010V was exchanged for a +1 D Staar AQ5010V, her postoperative refractive error improved to –0.75 +1.50 x 016, resulting in UCVA of 20/30+ and BCVA of 20/15-. Patient 12’s right eye could not be refracted before or after surgery because of severe myopic macular degeneration. This patient’s refractive outcome was not included in the analysis of IOL power calculation accuracy, but the patient was included in the overall outcomes analysis because he was one of the 12 implanted with a negative power IOL in this consecutive series. The IOLMaster was used to measure his axial length even though he was unable to fixate with the eye. He had a staphyloma, so we reasoned that immersion ultrasound would have been no more accurate.

## RESULTS

Demographic and surgical information are shown in Table **1**. The average patient in the study was younger (58.4 years) than the typical patient undergoing cataract surgery in this practice (73 years). More Caucasian eyes than Asian eyes were implanted. This is likely a reflection of the demographics of the clinical practice of one of the co-authors. IOLs from several different manufacturers were implanted. Four patients received negative power IOLs bilaterally. The mean follow up was 23.6 months.

Only patients with highly myopic eyes undergoing primary IOL implantation at the time of cataract surgery were included in this study. Patients receiving negative power piggyback and phakic IOLs were excluded.

Preoperative and postoperative visual acuities are shown in Table **2**. All patients had UCVAs of count fingers (CF) before surgery. Most did not achieve a BCVA of 20/20 or better following surgery because of pre-existing macular pathology. Even so, 6 eyes (37.5%) achieved 20/20 or better and 13 eyes (81.3%) achieved a BCVA of 20/40 or better, which is sufficient to obtain or maintain a driver’s license in most of the United States.

Axial lengths, manifest refractions, and SE refractive errors are shown in Table **3**. The mean axial length was 32.65 mm. Only one eye had an axial length shorter than 30 mm. The mean preoperative and postoperative SE refractive errors were –22.19 D and –0.21 D respectively. Eight of 15 eyes had hyperopic refractive errors after surgery although this was never the targeted postoperative refraction.

The postoperative SE refractive errors predicted by each IOL power formula and the refractive errors actually achieved are shown in Table **4**. The mean achieved postoperative SE refractive error was a little over 1 D more hyperopic than predicted by all 3 formulas. The difference between the mean intended and mean achieved SE refractive errors was +1.16 D for the SRK/T, +1.20 D for the Holladay 1, and +1.60 D for the Hoffer Q formulas. Only five (33.3%) of 15 eyes were within 1 D of the intended postoperative refraction. Twelve (80 %) of eyes were within 2 D of intended. The SRK/T formula, the one used to calculate IOL power for the patients in the study, proved to be the most accurate.

No intraoperative complications occurred. The majority of eyes developed visually significant posterior capsule opacification within a year of surgery. There were no postoperative retinal detachments.

## DISCUSSION

Before the advent of negative power intraocular lenses, extremely myopic eyes were usually left aphakic at the time of cataract surgery. Optical rehabilitation of the aphakic eye consisted of relatively low negative power spectacle or contact lenses to correct residual myopia. In our opinion it is preferable to implant an IOL. The advantages of pseudophakia over aphakia for these eyes include the possibility of an improved postoperative refractive status, stabilization of the anterior vitreous face to reduce the risk of postoperative retinal detachment, and the prevention of aphakic pupillary block.

Despite axial lengths of >30 mm in 15 of 16 eyes and the presence of myopic macular degeneration and/or posterior segment staphylomata in most eyes, all eyes experienced improvements in UCVA and BCVA. The improvements in UCVA were dramatic. The improvements in BCVA were also significant for many patients, probably owing to the density of the cataract before surgery and elimination of the image minifying effect of strong negative power glasses and contact lenses required before surgery.

Our patients were relatively young at the time of cataract surgery. This same observation that myopic eyes develop cataracts at a relatively young age was made by Hoffer in a biometric study of 7500 cataractous eyes [4]. We are unable to propose a mechanism for the premature cataract development.

In a study of 56 eyes that received negative power IOLs reported by Lin and colleagues, 36 (64.3%) of 56 eyes were 20/40 or better after surgery and 6 eyes (10.7%) were 20/20 or better [5]. In a study of 126 eyes reported by Ji and coworkers, 69 (54.8%) of 126 eyes were 20/40 or better after surgery [6]. Our postoperative BCVA results were slightly better than those reported in these two earlier studies.

Eight of 15 eyes that could be refracted experienced a hyperopic postoperative result in our study. This was never intended. The worst result was a postoperative SE refractive error of +1.75 D that prompted an IOL exchange. Seven of 15 eyes experienced a myopic result. The postoperative refractive error of patient 12 is unknown because it was not possible to obtain a reliable retinoscopy or auto-refraction after surgery. Xie and coworkers recommend targeting extremely myopic eyes for low myopia postoperatively [7]. We agree that this is the safest and most practical approach until the accuracy of IOL power calculation for these eyes improves. We recommend targeting highly myopic eyes for –1.5 D using the SRK/T formula if a negative power IOL is calculated and emmetropia or mild residual myopia is the desired postoperative result.

The most common complication in this series was posterior capsule opacification. This is the finding of other studies as well [5,6,8]. The reason for the high incidence is probably multifactorial. Most of the IOLs implanted did not have square edges. Additionally, capsular bags in extremely myopic eyes tend to be big and floppy and have striae connecting the apices of the haptics. At an average follow up of 23.6 months none of our patients experienced a retinal detachment. Most, if not all, had already experienced a posterior vitreous detachment. We could not find a single report of retinal detachment in any of the studies we reviewed [5,6,8,9]. Clearly a larger group and longer follow up are necessary to address this long-term concern, and an unoperated control group of eyes from age-matched patients would also be helpful for comparison purposes.

The SRK/T formula was the most accurate for the highly myopic eyes we studied, but with relatively few eyes in the study we cannot say that there is statistical significance in this finding (P=0.25). All three formulas predicted a postoperative refractive result that was more myopic than the one achieved. In other words, the achieved result was more hyperopic than intended, usually by more than 1 D.

The authors of one study found that the Hoffer Q formula was the most accurate for eyes with high axial myopia, resulting in the least unintended hyperopia [3]. This, however, is quite different from our conclusion. We found that the Hoffer Q formula was the least accurate formula for highly myopic eyes. This was shown by Dr. Hoffer himself [10]. All the studies we reviewed came to the conclusion that current methods of power calculation tend to produce hyperopic results as compared to the target refractive error when A-scan ultrasonography is used to measure axial length [2,3,9].

It is possible that the IOL power formulas are not completely at fault. Errors may also arise in axial length determination. The Humphrey Zeiss IOLMaster may prove to be more accurate for determining functional axial length in extremely myopic eyes because it measures the distance to the functional fovea. This test, of course, requires fixation for best results, which may not be present in eyes with severe myopic degeneration. Traditional A-scan ultrasonography, on the other hand, measures the longest axial length of the eye. For an eye with a staphyloma and a functional fovea or locus of best eccentric fixation on the slope of the staphyloma, A-scan ultrasonography will measure an anatomic axial length that is longer than the functional axial length. The visual acuity and fixation ability of the only patient in this series who was measured using the IOLMaster was too poor to validate the accuracy of the instrument. No matter which IOL power formula is used, if an excessively long axial length is measured it will calculate an IOL power that is too low, producing a hyperopic result.

To conclude, highly myopic eyes implanted with negative power IOLs at the time of cataract surgery did relatively well in this series in terms of visual and refractive outcomes. BCVA outcomes were limited by pre-existing macular pathology such as myopic retinal degeneration and staphyloma formation. Either the accuracy of axial length determination or the IOL power calculation or both limited refractive outcomes. The SRK/T formula was the most accurate when immersion A-scan ultrasound was used to measure axial length. We recommend adding +1.5 D to the power of the calculated lens to achieve the desired postoperative refraction. More work needs to be done to improve IOL power calculation accuracy in these eyes, and the Humphrey Zeiss IOLMaster may be the best tool to do it. Highly myopic eyes have a propensity for posterior capsule opacification, so patients should be counseled about the high likelihood they might need a laser capsulotomy after otherwise successful cataract surgery.

## Figures and Tables

**Table 1.** Demographic and Surgical Information of Patients Implanted with Negative Power Intraocular Lenses at the Time of Cataract Surgery

Patient	Age	Race	Sex	Eye	Date of Surgery	Lens Manufacturer and Model	Power (D)	Follow-Up (Months)	Postoperative Issues
1	40	C	F	R L	Dec 1996 Feb 1997	Storz P574UV Storz P574UV	-4 -3	48 47	S/P laser capsulotomy S/P laser capsulotomy
2	46	C	F	L	Jan 1998	Storz P517UV	-3	53	S/P laser capsulotomy
3	59	C	F	L R	Feb 1999 Sep 2002	Storz P517UV Alcon MA60MA	-6 -4	48 1	PCO
4	36	A	F	R	Nov 1999 Dec 1999	Staar AQ5010V Staar AQ5010V	-4 -4	46 45	PCO PCO
5	52	C	M	L R	Jan 2000 Feb 2000	Staar AQ5010V Staar AQ5010V	-2 -1	24 22	PCO
6	66	U	M	R	Jul 2000	Staar AQ5010V	-1	8	PCO
7	73	C	F	L	Jan-2001	Staar AQ5010V	-1	16	IOL exchange because of hyperopic postoperative refractive error; S/P laser capsulotomy
8	60	C	F	R	Apr 2001	Staar AQ5010V	-1	12	PCO
9	76	C	F	L	Oct 2001	Staar AQ5010V	-1	4	PCO
10	81	C	F	R	Feb 2002	Alcon MA60MA	-2	1	
11	53	A	M	L	Sep 2002	Alcon MA60MA	-1	1	
12	59	C	M	R	Apr 2002	Alcon MA60MA	-3	2	PCO
Mean	58.4							23.6	

D = Diopter, C = Caucasian, A = Asian, U = Unspecified, M = Male, F = Female, L = Left, R = Right, S/P = Status Post, PCO = Posterior Capsule Opacification (not requiring a capsulotomy), IOL = intraocular lens.

**Table 2.** Preoperative and Postoperative Visual Acuities of Eyes Implanted with Negative Power Intraocular Lenses at the Time of Cataract Surgery

Patient	Eye	Preoperative BCVA	Postoperative UCVA	Postoperative BCVA
1	R	20/200	20/30-3	20/15
	L	20/50	20/25-1	20/20
2	L	20/125-1	20/40+2	20/30+1
3	R	CF 1 foot	CF 4 feet	20/100-1
	L	20/125	20/125	20/40+2
4	R	20/160	20/50-2	20/30+2
	L	20/70	20/70-1	20/20-1
5	L	20/40-2	20/60+2	20/30+2
	R	20/40+1	20/25-2	20/20
6	R	CF 4 feet	20/60	20/30-1
7	L	20/30+2	20/40-2	20/15
8	R	CF 4 feet	20/50+2	20/20-1
9	L	CF 3 feet	20/125	20/125
10	R	20/40-1	20/40+1	20/30-2
11	L	CF 5 feet	20/50-1	20/30+1
12	R	CF 2 feet	20/300	20/300
Median		20/125-1	20/50-2	20/30+1

BCVA = Best Corrected Visual Acuity, UCVA = Uncorrected Visual Acuity, R = Right, L = Left, CF = Count Fingers.

**Table 3.** Axial Lengths, Manifest Refractions, and Spherical Equivalent Refractive Errors of Eyes Implanted with Negative Power Intraocular Lenses

Patient	Eye	Axial Length (mm)	Preoperative Manifest Refraction SE (D)		Postoperative Manifest Refraction SE (D)	
1	R	32.63	-30.00 +1.75 x 060	-29.13	+1.00 +0.50 x 176	+1.25
	L	32.65	-25.00 +0.50 x 165	-24.75	+0.50 +0.25 x 010	+0.63
2	L	31.70	-27.50 +1.00 x 063	-27.00	-1.00 +0.50 x 082	-0.75
3	L	35.60	-31.00 sphere	-31.00	-3.50 +0.50 x 059	-3.25
	R	34.36	-26.00 -2.00 x 025	-27.00	-1.25 +0.75 x 180	-0.87
4	R	31.41	-21.00 +0.50 x 138	-20.75	-1.50 +0.50 x 097	-1.37
	L	32.34	-22.25 +0.75 x 160	-21.87	-3.00 +1.00 x 120	-2.63
5	L	33.40	-23.00 sphere	-23.00	-2.00 +1.50 x 062	-1.25
	R	33.27	-20.00 sphere	-20.00	-1.50 +1.25 x 132	-0.75
6	R	33.01	-27.00 sphere	-27.00	+0.75 +0.25 x 180	+0.87
7	L	28.80	-15.25 +2.25 x 175	-14.13	+1.75 sphere	+1.75
8	R	30.33	-16.00 sphere	-16.00	+1.25 +0.25 x 174	+1.37
9	L	33.43	-15.75 +3.25 x 086	-14.13	+1.00 sphere	+1.00
10	R	31.15	-18.50 +0.75 x 075	-18.13	-0.25 +1.00 x 175	+0.25
11	L	32.53	-19.00 sphere	-19.00	Plano +1.25 x 016	+0.63
12	R	35.77	Unable to refract	N/A	Unable to refract	N/A
Mean		32.65		-22.19		-0.21

SE = Spherical Equivalent, D = Diopter, N/A = Not applicable.

**Table 4.** Postoperative Predicted and Achieved Spherical Equivalent Refractive Errors as a Function of Lens Power Formula following Cataract Extraction with Negative Power Intraocular Lens Implantation

Patient	Eye	Predicted SE (D) Refractive Error			Achieved SE (D) Refractive Error
		SRK/T	Hoffer Q	Holladay 1	
1	R	-0.49	-0.91	-0.44	+1.25
	L	-1.30	-1.75	-1.31	+0.63
2	L	-1.82	-2.12	-1.71	-0.75
3	L	-2.81	-3.09	-2.48	-3.25
	R	-2.45	-2.89	-2.38	-0.87
4	R	-1.13	-1.32	-0.89	-1.25
	L	-2.52	-2.76	-2.32	-2.50
5	L	-1.07	-1.66	-1.26	-1.25
	R	-1.15	-1.75	-1.40	-0.75
6	R	-1.15	-1.75	-1.40	+0.87
7	L	-0.87	-1.31	-1.04	+1.75
8	R	-0.94	-1.40	-1.08	+1.37
9	L	-0.78	-1.38	-1.04	+1.00
10	R	-1.27	-1.64	-1.27	+0.25
11	L	-0.87	-1.45	-1.11	+0.63
12	R	N/A	N/A	N/A	Unable
Mean		-1.37	-1.81	-1.41	-0.21

SE = Spherical Equivalent, D = Diopter, L = Left, R = Right, N/A = Not applicable.
